# Long-term clinical outcomes following treatment with alpha 1-proteinase inhibitor for COPD associated with alpha-1 antitrypsin deficiency: a look at the evidence

**DOI:** 10.1186/s12931-017-0574-1

**Published:** 2017-05-30

**Authors:** Franck F. Rahaghi, Marc Miravitlles

**Affiliations:** 1 Pulmonary and Critical Care Division, Cleveland Clinic Florida, 2950 Cleveland Clinic Blvd, Weston, FL 33331 USA; 2 Pneumology Department, Hospital Universitari Vall d’Hebron, Ciber de Enfermedades Respiratorias (CIBERES), Passeig de la Vall d’Hebron, 119-129, 08035 Barcelona, Spain

**Keywords:** Alpha-1 antitrypsin, Alpha-1 antitrypsin deficiency, Chronic obstructive pulmonary disease, Emphysema, Mortality, Treatment, Outcome

## Abstract

Alpha-1 antitrypsin deficiency (AATD) is a common hereditary disorder caused by mutations in the *SERPINA1* gene, which encodes alpha-1 antitrypsin (AAT; also known as alpha 1-proteinase inhibitor, A_1_-PI). An important function of A_1_-PI in the lung is to inhibit neutrophil elastase, one of various proteolytic enzymes released by activated neutrophils during inflammation. Absence or deficiency of A_1_-PI leads to an imbalance between elastase and anti-elastase activity, which results in progressive, irreversible destruction of lung tissue, and ultimately the development of chronic obstructive pulmonary disease with early-onset emphysema. AATD is under-diagnosed, patients can experience long delays before obtaining an accurate diagnosis, and the consequences of delayed diagnosis or misdiagnosis can be severe. Currently, A_1_-PI therapy is the only available treatment that addresses disease etiology in patients with AATD; however, demonstrating clinical efficacy of A_1_-PI therapy is challenging. In order to show therapeutic efficacy with traditional endpoints such as forced expiratory volume in one second and mortality, large sample sizes and longer duration trials are required. However, AATD is a rare, slow progressive disease, which can take decades to manifest clinically and recruiting sufficient numbers of patients into prolonged placebo-controlled trials remains a significant obstacle. Despite this, the Randomized, placebo-controlled trial of augmentation therapy in Alpha 1-Proteinase Inhibitor Deficiency (RAPID) and RAPID Extension trial, the largest clinical program completed to date, utilized quantitative chest computed tomography as a sensitive and specific measure of the extent of emphysema. Findings from the RAPID/RAPID Extension program definitively confirmed the benefits of A_1_-PI therapy in slowing disease progression and provided evidence of a disease-modifying effect of A_1_-PI therapy in patients with AATD. These findings suggest that the early introduction of treatment in patients with severe emphysema-related AATD may delay the time to death, lung transplantation or crippling respiratory complaints. In addition, there is now limited evidence that A_1_-PI therapy provides a gain of more than five life-years, supporting previous observations based on registry data. With the clinical efficacy of A_1_-PI therapy now demonstrated, further studies are required to assess long-term outcomes.

## Background

Alpha-1 antitrypsin deficiency (AATD) is a common hereditary disorder caused by mutations in the *SERPINA1* gene, which encodes alpha-1 antitrypsin (AAT; also known as alpha 1-proteinase inhibitor, A_1_-PI) [1]. Mutations within this gene can lead to the production of a misfolded protein that accumulates in the endoplasmic reticulum, resulting in reduced secretion, and hence reduced circulating levels, of A_1_-PI [2]. In addition, variants can lead to a complete absence of protein product (null/null genotypes) or the production of normal levels of a dysfunctional protein (e.g., the F variant, which is associated with decreased binding to neutrophil elastase) [3]. An important function of A_1_-PI in the lung is to inhibit neutrophil elastase, one of various proteolytic enzymes released by activated neutrophils during inflammation. Therefore, absence or deficiency of A_1_-PI leads to an imbalance between elastase and anti-elastase activity. This results in progressive, irreversible destruction of lung tissue, and ultimately the development of chronic obstructive pulmonary disease (COPD) with early-onset emphysema, which increases the risk of crippling respiratory complaints and premature death [4].

Between 2007 and 2010, the number of at-risk individuals in the USA who were tested for AATD doubled [5]. Despite this, AATD is under-diagnosed, and of the estimated 100,000 Americans thought to be affected, fewer than 10% have been diagnosed [6]. Therefore, there is a need to identify symptomatic patients who may benefit from A_1_-PI therapy. In addition, patients with AATD are often misdiagnosed. Respiratory symptoms, including coughing, wheezing, dyspnea and excessive sputum production [7], are also common to other respiratory conditions such as smoking-related COPD or asthma. As a result, patients can experience long delays before obtaining an accurate diagnosis, with average delays of 6 years being reported [8].

The consequences of delayed diagnosis or misdiagnosis can be severe. In addition to lung complications, AATD is associated with liver damage; clinical presentation is variable but ranges from neonatal hepatitis to liver cirrhosis and hepatocellular carcinoma in adults [9]. Currently, for severe liver disease, there is no known treatment beyond liver transplantation, and for selected patients with end-stage lung disease, lung transplantation is a valuable treatment option. If not treated by lung transplant, patients with AATD and a forced expiratory volume in one second (FEV_1_) below 20% predicted have a 2-year mortality of 40% [10]. Between 2011 and 2014, the median waiting list time for a lung transplant in the US was 226 days (95% confidence interval: 187, 268) for patients aged 35–49 years (last available data from the Organ Procurement and Transplantation Network) [11]. In May 2005, a new system was developed [12], and for the majority of patients, its introduction has reduced the time to lung transplant and the risk of death whilst on the waiting list [13].

Other less common clinical manifestations of AATD include: panniculitis, a serious skin condition [14]; and airway disease such as bronchiectasis [15]. AATD is also associated with vasculitis, an inflammation of blood vessel(s) [16]. With increased disease awareness, early diagnosis and optimal management (e.g., lifestyle changes such as smoking cessation and exercise, and the institution of effective therapy), it may be possible to lessen and/or delay the onset of these deleterious health consequences.

Currently, A_1_-PI therapy is the only available treatment that addresses disease etiology in patients with AATD. Historical registry data suggested a mortality benefit of A_1_-PI, in addition to a benefit on FEV_1_ decline in a subgroup of patients with moderate-to-severe baseline FEV_1_ impairment [17]. Furthermore, a meta-analysis of five trials observed a 23% slower decline in FEV_1_ among patients receiving A_1_-PI therapy [18]. However, initial clinical trials failed to demonstrate a significant benefit in reducing the rate of decline in lung function but their results were suggestive of some protection against a loss of lung tissue [19, 20]. More recently, the Randomized, placebo-controlled trial of augmentation therapy in Alpha 1-Proteinase Inhibitor Deficiency (RAPID) and RAPID Extension trial, the largest clinical program completed to date, definitively confirmed the benefits of A_1_-PI therapy in slowing disease progression [21, 22].

This narrative review discusses the relevance of outcome measures to assess AATD-related emphysema progression, and mortality data from previous studies of A_1_-PI therapy. In addition, an overview of the RAPID/RAPID Extension program will be provided and relevant *post-hoc* findings from will be examined. Data were sourced from a literature review conducted on MEDLINE with no language or time period settings applied. Search terms included US National Library of Medicine Medical Subject Headings (MeSH) such as ‘alpha-1 antitrypsin deficiency’ and ‘alpha-1 antitrypsin’ and ‘mortality’. In addition, the reference lists of sourced articles were checked, and the author’s personal literature collection was utilized.

### Outcome measures and mortality prediction in patients with AATD

COPD is a progressive condition characterized by airflow obstruction that is not fully reversible [23]. Pathological changes can be found in the central and peripheral airways, lung parenchyma and pulmonary vasculature [23]. The destruction of the lung parenchyma or pulmonary emphysema is associated with abnormal permanent enlargement of the distal airspaces beyond the terminal bronchioles accompanied by destruction of the alveolar walls, and without obvious fibrosis [6]. It is well established that AATD is associated with early-onset emphysema; in addition, the presence of airway disease, including a high prevalence of bronchiectasis and bronchial wall thickening, has been reported [15]. In AATD, emphysema is predominantly distributed in the basal region of the lung and is associated with a greater degree of airflow obstruction. This is in contrast to the impairment of gas exchange typically seen with emphysema distributed in the apical zone [24].

The early identification of these pathological changes is key to preventing widespread destruction of the lung parenchyma. FEV_1_ has been traditionally regarded as the ‘gold standard’ endpoint for the assessment and monitoring of obstructive airways disease, and has been proposed as a potential surrogate marker for emphysema progression. However, FEV_1_ lacks the sensitivity to effectively chart disease progression in patients with AATD. Changes in FEV_1_ occur slowly and unevenly over time. Therefore, demonstrating a therapeutic benefit would require a placebo-controlled trial of at least 5 years duration, and the recruitment of a minimum of 1000 subjects [25]. Compared with usual COPD [26], the recruitment of sufficient numbers of patients for clinical trials of rare diseases such as AATD is difficult. Furthermore, emphysema can be present in usual COPD and AATD even when FEV_1_ is in the normal range [24, 27].

Despite its limitations, FEV_1_ has been used to stratify disease severity in COPD in general, and mortality risk in patients with AATD in particular. Findings of an early National Heart, Lung, and Blood Institute (NHLBI) registry study of patients with severe AATD (*n* = 1129) showed that, compared with patients not receiving active treatment, the use of A_1_-PI therapy was associated with lower mortality (risk ratio = 0.64, 95% CI: 0.43 to 0.94, *p* = 0.02) [17]. This benefit was particularly evident in those patients with a baseline FEV_1_ % predicted below 50%. More recent analysis of these data further stratified patients based on their baseline FEV_1_ levels and other lung function parameters. A_1_-PI therapy was shown to be associated with improved survival in patients with baseline FEV_1_ % predicted <20% (*n* = 161), <30% (*n* = 424) and 30–65% (*n* = 444; *p* < 0.0001 for all); a benefit that was not observed in patients with baseline FEV_1_ % predicted >65% (*n* = 258; *p* = 0.38) [28]. Nevertheless, for a clinical study using mortality as a primary endpoint, it has been estimated that 684 individuals with AATD and a baseline FEV_1_ of 35–49% predicted would need to be followed in a 5-year study to detect a 40% reduction in mortality [29].

In 2009, the US Food and Drug Administration (FDA) approved computed tomography (CT) as an appropriate clinically meaningful endpoint to assess the efficacy of therapy with intravenous A_1_-PI products on emphysema disease progression, and permitted its use as a primary endpoint in Phase IV studies [30]. This approval is based on studies of patients with emphysema that show correlations between lung density, as measured by high resolution CT, and anatomic pathology, pulmonary function tests, and mortality [30]. CT densitometry not only provides information regarding overall lung destruction, it establishes the specific locations in the lung where the emphysematous destruction has occurred, and is now recognized as the most specific and sensitive outcome measure for assessing the progression of emphysema [20, 31–33]. Percentile density 15 (PD15), defined as the density value (Hounsfield Unit) at which 15% of the voxels in the frequency distribution histogram have a lower density, is a sensitive index of change in lung density. In a group of patients with a wide range of lung function impairment, PD15 was shown to be 2.5-fold more sensitive than currently recommended lung function parameters for the measurement of emphysema progression, including spirometry and carbon monoxide gas transfer [33]. Furthermore, in contrast to FEV_1_, the decline in PD15 is linear, which makes it a more consistent measure across the broad spectrum of disease severity seen in patients with AATD [20, 31].

**Fig. 1:**
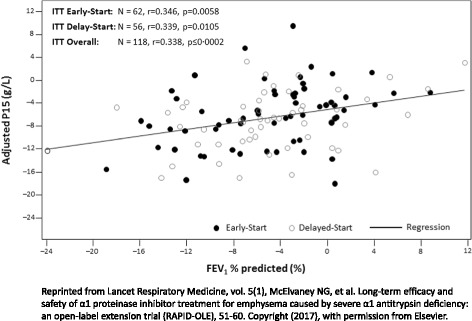
Correlation between changes in adjusted PD15 at TLC and changes in FEV1% predicted from Day 1 to Month 48 in the RAPID/RAPID Extension program (RAPID Extension ITT population). Adjusted PD15, lung volume-adjusted 15^th^ percentile of the lung density; FEV_1_, forced expiratory volume in one second; ITT, intention-to-treat; TLC, total lung capacity

The rate of decline in CT lung density has been shown to correlate with FEV_1_ decline [20–22, 31]. During the RAPID/RAPID Extension program, moderate-to-weak 4-year correlations were detected between decline in rate of lung density and spirometry values. The overall correlation coefficient was *r* = 0.338 for FEV_1_ % predicted (*p* < 0.001; Fig. 1) [22]. Despite this, a decline in CT lung density is not always reflected by a corresponding decline in FEV_1_. A study of 110 patients from a UK AATD registry found that approximately 50% of patients who showed evidence of lung density decline by CT had no significant evidence of FEV_1_ decline greater than natural aging [34]. However, some of these patients showed a decline in gas transfer and diffusing capacity of the lungs for carbon monoxide (DL_CO_), indicating that change in physiological measurements varies with their baseline values, whereas the change in PD15 does not. A 30 month longitudinal study across five centers indicates that changes in lung density, FEV_1_ and DL_CO_ may also occur independently of the levels measured at baseline [33].

In line with its greater sensitivity as a measure of emphysema progression, CT scanning has also been shown to be superior to lung function parameters, especially FEV_1_, for predicting mortality in patients with AATD [35]. Furthermore, results suggest that for patients with AATD, a change in basal lung density may be a better surrogate outcome measure for long-term mortality, than changes in either apical or whole lung density [34]. However, given the heterogeneity in the distribution pattern of emphysema in patients with usual or AATD-related COPD this may not be true for all patients. In AATD, although the emphysema has a predominantly basal distribution pattern, in approximately one third of patients a greater involvement of the apical regions is seen [24]. To date, whole lung density at full inspiration (similar to total lung capacity [TLC]) is the measure that has been commonly used in randomized controlled trials (RCTs) of A_1_-PI therapy [19–22].

Studies have also evaluated clinical- and patient-reported outcome measures to assess therapeutic efficacy, including health status and mortality. It is well established that AATD-related emphysema impacts on a patient’s quality of life (QoL), and that this occurs at a younger age than non-AATD-related COPD [36, 37]. Studies have highlighted that exacerbations contribute significantly to impaired QoL in patients with AATD [38, 39]. Preliminary data from a patient survey and a small retrospective epidemiological study, suggest that A_1_-PI therapy may reduce the incidence and severity of exacerbations. [40, 41]. A *post hoc* analysis of data from the EXAcerbations and Computed Tomography scan as Lung End-points (EXACTLE) trial indicated that although augmentation therapy was associated with a reduction in exacerbation severity, it did not alter exacerbation frequency [20]. However, further evaluation in a larger placebo-controlled trial is still lacking.

### New evidence from RAPID/RAPID Extension program

The feasibility of A_1_-PI therapy for the treatment of AATD-associated emphysema was first demonstrated in the 1980s [42, 43]. Infusion of A_1_-PI was shown to increase plasma levels above the protective threshold level (>11 μM) and restore the protease-antiprotease balance within the lung. However, the Danish-Dutch [19] and the EXACTLE [20] trials were the first RCTs to assess the efficacy of A_1_-PI therapy to prevent the progression of emphysema in patients with AATD. Although neither trial reported a significant effect of A_1_-PI therapy on pulmonary function, CT analysis showed a trend towards a more favorable effect of treatment, which was suggestive of lung tissue preservation compared with placebo. Furthermore, a combined analysis of both trials confirmed a significant reduction in the decline in lung density with A_1_-PI therapy [44].

The RAPID/RAPID Extension program, in addition to being the largest trial of A_1_-PI therapy in patients with AATD completed to date, is the only trial specifically designed to assess the disease-modifying effect of treatment. [45] The program consisted of the 2-year, randomized, double-blind, placebo-controlled study (RAPID-RCT) followed by the 2-year open-label extension study (RAPID-OLE). The primary endpoint was annual rate of decrease in lung density as assessed by adjusted PD15. During RAPID-RCT, the annual rate of lung density loss at TLC alone was significantly lower in patients receiving A_1_-PI therapy compared with placebo (*n* = 180; −1·45 g/L/year vs. –2·19 g/L/year; *p* = 0.017, one-sided test); corresponding to a 34% reduction in the progression of emphysema in favor of A_1_-PI therapy [21, 22]. For the first time since clinical research began in this area in the 1980s, the efficacy of A_1_-PI therapy, as evaluated by an FDA endorsed endpoint, was demonstrated [30].

This beneficial effect was confirmed in RAPID-OLE in which all patients received A_1_-PI therapy. Overall, from Day 1 to Month 48, treatment differences were shown to favor the Early-Start group (i.e., patients who received A_1_-PI treatment during RAPID-RCT), substantiating continued efficacy over 4 years. During RAPID-OLE (Month 24 to Month 48), the rate of decline in lung density was similar between the Early-Start and Delayed-Start groups (i.e., patients who received placebo during RAPID-RCT) [22]. Furthermore, a statistically significant reduction in the annual rate of lung density decline was established in the Delayed-Start group following the switch from placebo administration to active therapy (a difference of 0.52 g/L/year; *p* = 0.001) [22]. However, the loss of lung density that occurred in the Delayed-Start group prior to receiving A_1_-PI therapy was not regained with the initiation of active treatment [22]. Taken together these findings demonstrate a disease-modifying effect of A_1_-PI therapy in patients with AATD.

Overall, between Day 1 and Month 48, no significant differences were observed between treatment groups in secondary endpoints such as quality of life (St George’s Respiratory Questionnaire score) or pulmonary function tests, including FEV_1_ [21, 22]. The authors suggest this is not surprising since the trial was not designed with sufficient power to detect such changes.

The rates of decline in lung density in patients with AATD as seen in the RAPID/RAPID Extension program are higher than those reported in other trials of lung-related conditions. In patients with COPD, and a mean baseline post-bronchodilator FEV_1_ % predicted of 48.5%, the rate of decline in lung density was variable with a mean decline of −1.13 g/L/year [46]. The decline in lung density was more rapid in women than men, and in current smokers than former smokers. Furthermore, in patients diagnosed with emphysema by high-resolution CT scan, and a mean baseline FEV_1_ % predicted of 50.2%, the annual rate of lung density decline was −1.31 g/L/year [33].

### RAPID/RAPID Extension life-years gained analysis

**Fig. 2:**
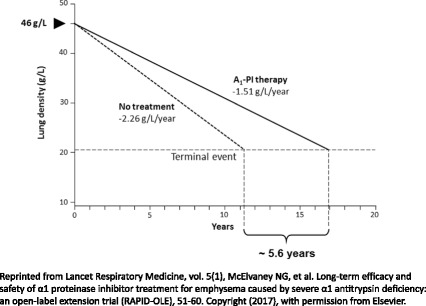
Estimated time to terminal respiratory failure based on data from RAPID/RAPID Extension program. Based on lung volume-adjusted 15^th^ percentile of the lung density. A_1_-PI, alpha 1-proteinase inhibitor

A *post-hoc* analysis of the RAPID/RAPID Extension program investigated the time to respiratory crisis for progressive emphysema, defined as death, lung transplant or a crippling respiratory condition. Seven patients withdrew from the 4-year program (final mean FEV_1_ % predicted of 36.1%) due to a respiratory crisis, with an average final recorded lung density of approximately 20.5 g/L (Table 1). Using this value as the terminal lung density, and by comparing the rate of decline in lung density loss in A_1_-PI-treated patients (−1.51 g/L/year) with placebo-treated patients (−2.26 g/L/year), the projected gain in life-years (time to terminal respiratory failure) was approximately 5.6 years (Fig. 2) [21, 22].

**Table 1:** Patient data for life-years gained analysis

Subject	Treatment Arm	Last AE term	Reason for discharge	Age at randomization	Sex	Baseline FEV_1_ % predicted	Baseline lung density (g/L)	Last measured lung density (g/L)	Time from the study start date to last measured lung density (years)
RAPID-RCT									
1	Placebo	Chest infection	Lung transplantation	57	M	44.9	6.5	3.5	0.40
2	A_1_-PI	Dyspnea	Withdrawal by patient	50	F	26.7	14.3	12.5	1.11
3	Placebo	Pulmonary infiltration	Withdrawal by patient	56	M	34.5	31.3	29.4	1.11
4	A_1_-PI	Severe respiratory insufficiency	Death	58	F	35.3	25.1	25.1	0.00
5	A_1_-PI	UTI	Lung transplantation	55	M	62.6	22.8	24.3	0.25
RAPID-OLE									
6	A_1_-PI (Early-Start)	Cross reactivity to birch allergy	Death	56	M	40.2	35.1	25.7	2.99
7	A_1_-PI (Delayed-Start)	Mycobacteria infection	Lung transplantation	48	F	33.0	32.7	23.2	3.94

*A*_*1*_ *-PI* alpha 1-proteinase inhibitor, *AE* adverse event, *F* female, *FEV*_*1*_ forced expiratory volume in one second, *M* male, *RAPID-OLE* 2-year open-label RAPID Extension trial, *RAPID-RCT* 2-year, placebo-controlled RAPID trial, *UTI* urinary tract infection

This *post-hoc* calculation has a number of limitations. The calculation is based on a very small sample size (*n* = 7), and is only a preliminary estimate. In addition, the analysis presumes a linear decline in loss of lung density. Furthermore, whilst the analysis aimed to explore a mortality benefit, the trials in the RAPID program were not sufficiently powered to assess mortality [21]; a substantially larger pool of terminal CT data from patients with AATD, similar to that of the NHLBI registry (*n* = 1129), would be needed to refine and validate this approach. Three deaths were recorded in the placebo arm of RAPID-RCT compared with one death in the active treatment arm over the first two years. An additional death was reported in the Early-Start group of the 2-year RAPID-OLE.

However, the life-years gained prediction is supported by historical survival improvement observations based on NHLBI Registry data [17]. In addition, these findings are further supported by Green et al., who showed that the improvement in the rate of lung-density decline provided by A_1_-PI therapy is greater than the difference between the whole-lung densities of those who died and those who survived [34]. The authors also state that it is unsurprising a mortality difference has not been observed in clinical trials, as their Kaplan Meier plots indicated that deaths occurred in the longer term (>3 years) [34]. Even clinical trials in smoking-related COPD, which are able to recruit much larger patient populations than rarer diseases such as AATD, have thus far been insufficiently powered to uncover differences in mortality. The TOwards a Revolution in COPD Health (TORCH) study, which included 6112 patients and followed them for 3 years, failed to show a reduction in death from all causes among patients with COPD in the combination therapy group (50 μg salmeterol plus 500 μg fluticasone propionate, twice daily) compared with those on placebo (*p* = 0.052) [47].

### Clinical context

Evidence is beginning to indicate an improvement in survival in patients with AATD receiving A_1_-PI therapy. It has also been suggested that a reduction in the progression of emphysema with A_1_-PI therapy may reduce the deterioration in health status [44]. In the RAPID/RAPID Extension program, patients in the Delayed-Start group who switched to active treatment did not regain the lung tissue they lost while on placebo. This highlights the importance of early intervention with A_1_-PI therapy in order to minimize and/or delay the onset of the deleterious health consequences associated with AATD.

However, as with other chronic illnesses, A_1_-PI therapy may pose a considerable burden on patients, their lifestyle, their families, and the healthcare system. Depending on healthcare structure, weekly intravenous administration of A_1_-PI may require a long journey to the hospital or the treating physician responsible for administering the treatment. This inconvenience could be addressed in part by home-treatment, whereby the infusion is administered by a visiting nurse [48] or the patient themselves. In fact, self-infusion is covered by the drug label for some of the licensed drugs used to treat patients with AATD [49]. Another approach could be to extend the infusion intervals to bi-weekly infusions of a double dose, or even 3-weekly infusions of a triple dose, as recommended in the Spanish treatment guidelines [50]. Extended infusion intervals are not covered by the drug labels; however, available clinical data, and data from RAPID-RCT, indicate that there are no safety issues with this approach [51].

In contrast to hemophilia therapies, where plasma-derived products have in part been replaced by recombinant products, no recombinant A_1_-PI is currently available. While intermittent shortage of drug supply was an issue in the late 1990s and early 2000s, the increasing number of companies with licensed A_1_-PI products has helped to eliminate this problem. However, as with many treatments requiring plasma products or solely produced for the treatment of rare diseases it comes at a cost.

In the long-term, A_1_-PI therapy has the potential to offer benefits with the life-years extension in comparison with the high mortality and morbidity rates associated with lung transplantation. Between January 1994 and June 2011, adults who underwent either a single of double lung transplantation (*N* = 37,581), which included patients with COPD (34%) and AATD (6%), had a median survival of 5.6 years [52]. Survival rates for lung transplantation vary by indication, and the presence of AATD has been shown to be an independent predictor of 1-year mortality [52]. In addition, a trend towards worse early post-lung transplantation survival has been shown in patients with AATD compared with those without [53]. In patients with severe AATD, and end-stage lung disease, those patients who received a lung transplant had significantly improved long-term survival compared with those who did not (estimated median survival time 11 years vs. 5 years, respectively; *p* = 0.006) [54]. However, it should be noted that no patients in the control group received A_1_-PI therapy.

Further study and validation of the terminal lung density threshold used in the life-years gained analysis is required. It would allow better planning for transplantation in patients with AATD, and is also important in terms of disease prognosis and life planning, and perhaps even the decision to treat.

## Conclusions

Demonstrating clinical efficacy of A_1_-PI therapy is challenging. In order to show therapeutic efficacy with traditional endpoints such as FEV_1_ and mortality, large sample sizes and longer duration trials are required. However, AATD is a rare, slow progressive disease, which can take decades to manifest clinically [55], and recruiting sufficient numbers of patients into prolonged placebo-controlled trials remains a significant obstacle. Despite this, the RAPID/RAPID Extension program has provided evidence of a reduction in the rate of lung density decline and a disease-modifying effect of A_1_-PI therapy in patients with AATD [21, 22], with possible implications for long-term changes in mortality. These findings suggest that the early introduction of treatment in patients with severe emphysema-related AATD may delay the time to death, lung transplantation or crippling respiratory complaints. Further studies are required to assess long-term outcomes.

## Abbreviations

A_1_-PI: Alpha 1-proteinase inhibitor
AAT: Alpha-1 antitrypsin
AATD: Alpha-1 antitrypsin deficiency
COPD: Chronic obstructive pulmonary disease
CT: Computed tomography
DLco: Diffusing capacity of the lungs for carbon monoxide
EXACTLE: EXAcerbations and Computed Tomography scan as Lung End-points
FDA: US Food and Drug Administration
FEV_1_: Forced expiratory volume in 1 s
LAS: Lung allocation score
MeSH: US National Library of Medicine Medical Subject Headings
NHLBI: National Heart, Lung and Blood Institute
PD15: Percentile density 15
QoL: Quality of life
RAPID: Randomized, placebo-controlled trial of augmentation therapy in Alpha 1-Proteinase Inhibitor Deficiency
RAPID-OLE: 2-year open-label RAPID Extension trial
RAPID-RCT: 2-year, placebo-controlled RAPID trial
RCT: Randomized controlled trial
TLC: Total lung capacity
TORCH: TOwards a Revolution in COPD Health.
